# Research progress in *in vivo* tracing technology for extracellular vesicles

**DOI:** 10.20517/evcna.2023.49

**Published:** 2023-12-07

**Authors:** Yanhua Shi, Xianghui Wang, Shifang Zhang, Hao Yin, Huaju Fan, Yaohui Tang, Nana Yang

**Affiliations:** ^1^School of Bioscience and Technology, Weifang Medical University, Weifang 261053, Shandong, China.; ^2^Medical Laboratory Animal Center, Weifang Medical University, Weifang 261053, Shandong, China.; ^3^Weifang Key Laboratory of Animal Model Research on Cardiovascular and Cerebrovascular Diseases, Weifang 261053, Shandong, China.; ^4^School of Rehabilitation Medicine, Weifang Medical University, Weifang 261053, Shandong, China.; ^5^School of Psychology, Weifang Medical University, Weifang 261053, Shandong, China.; ^6^School of Biomedical Engineering and Affiliated Sixth People’s Hospital, Shanghai Jiao Tong University, Shanghai 200030, Shanghai, China.; ^#^Authors contributed equally.

**Keywords:** Extracellular vesicles, *in vivo* tracing, molecular imaging, multimodal

## Abstract

Cells have the capability to discharge extracellular vesicles (EVs) into a range of bodily fluids. Extracellular vesicles (EVs) encapsulate biological molecules such as proteins and nucleic acids, playing a role in facilitating cell-cell communication. They actively engage in a myriad of physiological and pathological processes. *In vivo* tracing of EVs in organisms significantly contributes to elucidating the biological mechanisms of EV-based therapy. The development of molecular imaging technology makes it possible to trace EVs *in vivo*. Experiments frequently employ a range of molecular imaging techniques, encompassing bioluminescence imaging, fluorescence imaging, magnetic resonance imaging, single photon emission computed tomography, positron emission tomography, photoacoustic imaging, and multimodal imaging. These methods have their own advantages and disadvantages. In this review, typical applications of *in vivo* tracing of EVs are reviewed.

## INTRODUCTION

Extracellular vesicles (EVs) are a group of nanoscale heterogeneous lipid bilayer membrane vesicles, which are released into the extracellular environment by different cell types through various stages of evolution^[1]^. EVs can be detected in a range of liquid body substances, including but not limited to blood, breast milk, urine, and saliva^[2-5]^.

EVs were first observed in healthy human plasma by Chargaff *et al.* in 1946. Initially, they were recognized as a class of platelet microparticles with procoagulant effects^[6]^.

In 1967, Wolf first utilized electron microscopy to examine the structure and dimensions of these particulate deposits and named them “platelet dust”^[7]^. Subsequently, Johnstone *et al.* first formalized the name of this granular material as exosomes in an *in vitro* culture study of sheep reticulocytes^[8]^. In 2011, György *et al.* proposed to refer to all the different types of lipid bilayer-encapsulated extracellular structures as “extracellular vesicles (EVs)”^[9]^.

Initially, EVs were considered primarily as a means for cells to expel waste substances, and for an extended period, they remained relatively overlooked. Over the last several years, with the deepening of research, it has gradually been discovered that EVs contain other bioactive substances such as mRNA, ncRNA, miRNA, lncRNA, protein, and DNA, and have a lipid structure of information carriers, which can be used as transmitters of genetic information^[10]^. The surface molecules of EVs allow them to specifically target and reach receptor cells. Once attached to the target cell, EVs can activate receptor-ligand interactions or fuse with the target cell membrane through endocytosis, delivering their contents into the cytoplasm and thereby altering the physiological state of the receptor cell^[11]^. Diseases such as cancer can be predicted or diagnosed by detecting the presence and number of specific EVs; thus, EVs can be employed as a valuable diagnostic marker in the identification of diseases. In addition, EVs can be used as drug delivery agents. By utilizing EVs as carriers, drugs can be accurately delivered to the lesion site, thereby increasing the efficacy of the drug and reducing side effects.

According to the different sizes, biological properties, and formation processes, EVs are primarily categorized into three distinct groups: exosomes, microvesicles (MVs), and apoptotic bodies^[12,13]^. Exosomes emerge through the fusion of vesicles with cell membranes, exhibiting sizes ranging from 30 to 150 nm^[14]^. MVs are generated by the budding process of cell membranes and typically have a diameter spanning from 50 to 1,000 nm^[15]^. Apoptotic bodies are vesicles formed by cell shrinkage and fragmentation in the process of cell apoptosis, with a diameter of 500-2,000 nm^[16]^. Among them, exosomes have received more attention due to their unique generation pathway.

Studies have found that different cell types or different physiological states can cause expression differences in exosome contents. For example, exosomes derived from B cells contain B cell receptors (BCR), and exosomes derived from DC cells contain CD86, MCH-II, ICAM-1 proteins, *etc.*^[17]^. However, exosomes usually contain some common specific proteins that can be used as their markers, such as the adhesion molecule MFGE8^[18]^, the proteins involved in membrane transport such as Annexins, Flotillins and GTPases, and the proteins engaged in the creation of the multivesicular body (MVB) encompass Alix, TSG101, and Clathrin. Additionally, four transmembrane proteins, namely CD9, CD63, CD81, and CD82, as well as heat shock proteins such as HSP90, HSP70, HSP60, and HSPA5, play crucial roles in these cellular processes^[18-21]^. Kowal confirmed that the protein in exosomes is different from other subtypes of vesicle structure using protein profile analysis^[22,23]^. Lundholm also reported that the proteins in exosomes are different from cellular structural protein components, such as cell membranes, cytoplasm, mitochondria, Golgi, and endoplasmic reticulum^[24]^. These data prove that exosomes are a special type of subcellular structure, which is different from cell fragments.

Currently, the most extensively studied subgroups are exosomes and MVs. However, there is some overlap in diameter size between MVs and exosomes. So far, due to the lack of specific markers and technical methods, there has not yet been a strategy to unequivocally separate these two types of EVs in complex biological fluid samples^[25,26]^. Therefore, EVs is uniformly used as a generic term in this review.

**Figure 1:**
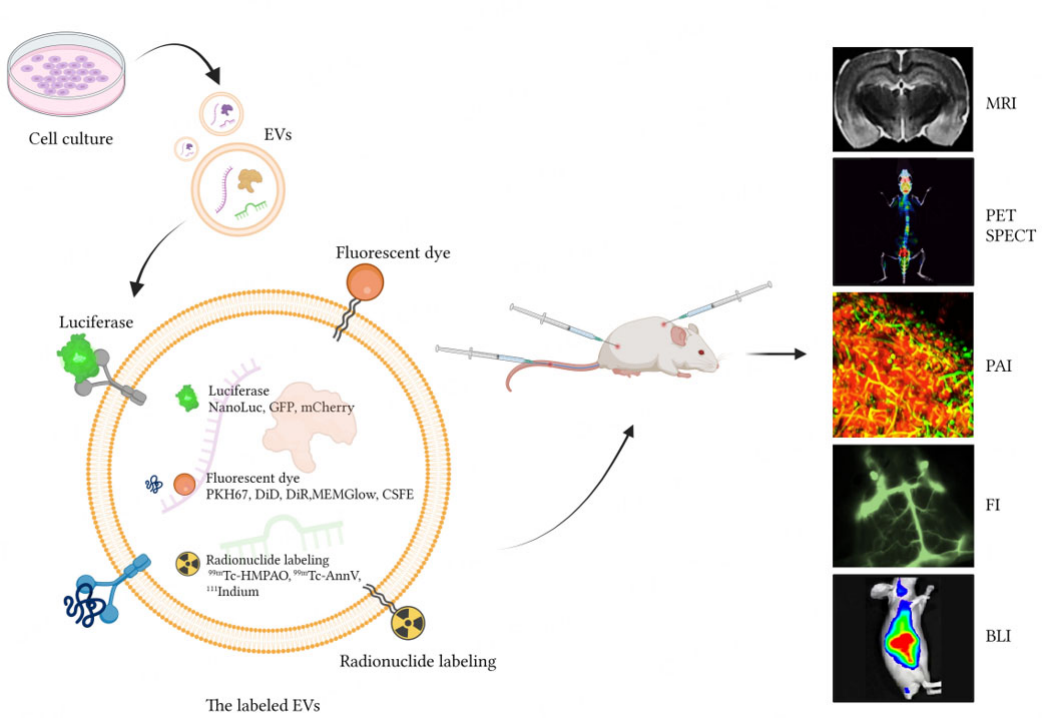
Strategies for tracking extracellular vesicles *in vivo*. Extracellular vesicles (EVs) can be extracted from the culture medium of donor cells or genetically modified cells by centrifugation. EVs can be modified to contain luciferase (NanoLuc, GFP, mCherry), fluorescent dyes (PKH67, DiD, DiR, MEMGlow, CSFE), and radionuclide labeling (^99m^Tc-HMPAO, ^99m^Tc-AnnV, 111Indium) for injection into animals for easy tracking. There are many imaging methods that can be used to track EVs *in vivo*, such as bioluminescence imaging (BLI), fluorescence imaging (FI), magnetic resonance imaging (MRI), positron emission tomography (PET), single photon emission computed tomography (SPECT), and photoacoustic imaging (PAI). Therefore, the *in vivo* tracing strategy of EVs is a key means to elucidate the biological mechanism of EV therapy. Created By Biorender.

Recently, the study of EVs has been deepened with the continuous development of biological and medical research. In order to monitor the activities of EVs in organisms more intuitively and in real time, and to observe the biological processes such as the production, transfer and distribution of EVs in living animals, researchers have gradually expanded the scope of research from *in vitro* to *in vivo*. This shift not only contributes to a more comprehensive understanding of the biological functions of EVs, but also presents fresh viewpoints and innovative ideas to enhance the understanding and management of diseases. The method of tracing EVs *in vivo* is to use specific imaging methods to track EVs in animals, so as to observe the biological behavior of EVs in the body. *In vivo* tracing of EVs was widely used because of its intuitive results, high sensitivity, and non-invasive nature (i.e., obtaining information or data of EVs through methods that do not cause any damage or interference to EVs). Several imaging methods are currently available for tracking EVs *in vivo*, such as bioluminescence imaging (BLI), fluorescence imaging (FI), magnetic resonance imaging (MRI), positron emission tomography (PET), and single photon emission computed tomography (SPECT), and photoacoustic imaging (PAI). The main current methods discussed in this review are succinctly summarized in Figure 1 and Table 1.

**Table 1:** EVs labeling and *in vivo* tracking strategies

**Imaging modality**	**Isolated sites/cells**	**Isolation method**	**Labeling agent**	**Concentration**	**Subject**	**Injection site**	**Imaging time points**	**References**
BLI	Prostate cancer cells (PC3)	UC	EGFP-CD63-Antares2	1 × 10^6^ cells/100 µL	BALB/c-nu/nu mice	Subcutaneously transplanting	0 d, 5 d, 10 d, 15 d, 20 d, 25 d, 30 d, 35 d	Hikita *et al.*^[28]^ (2020)
	HEK-293T cells/MSC cells	TFF/SEC/UC	DiR,CD63-NanoLuc/CD63-ThermoLuc	1 × 10^11^ cells/100 µL PBS	NMRI mice	IV	5 min, 15 min, 30 min, 60 min, 6 h, 24 h	Gupta *et al.*^[29]^ (2020)
	Plasma/cardiomyocyte/cardiac fibroblats	Exoquick^TM^ precipitation/ UC	CD63-NanoLuc	-	TG-αMHC-STOP-CD63NanoLuc mice	IP	5 min	Luo *et al.*^[30]^ (2020)
	Fibrosarcoma cells(HT1080)	Iodixanol gradient/UC	pHluo_M153R-CD63	1 × 10^6^ cells/mice; 1 × 10^5^ cells/chick	NOD/SCID mice, ex ovo chicks	Mouse mammary fat pad injection/IV	1 week, 24 h	Sung *et al.*^[31]^ (2020)
FI	Milk and cancer cells (U87, B16F10)	UC	SCy 7.5 and BDP-FL succinimidyl ester fluorophores	25 μg/150 μL PBS	AlbCre mice, mT/mG mice	IV	1 h, 4 h, 24 h	González *et al.*^[39]^ (2021)
	MSC	UC	DiR	100 µg/200 µL PBS	C57BL/6J mice(ischemic stroke)	IV	0 d, 1 d, 3 d, 5 d, 7 d, 10 d, 14 d	Xu *et al.*^[40]^ (2020)
	A549 cells	UC	DBCO-Cy5, DiD	1.5 mg/kg	Tumor-bearing mice	IV	24 h	Song *et al.*^[41]^ (2020)
MRI	CESC	UC	PKH67, DIR	40 μg/mL	SD rats (CEP and IVDD)	IV	6 weeks	Luo *et al.*^[43]^ (2021)
	MSC	UC	FTH1	50 µg/100 µL PBS	C57BL/6 mice	IM	After injection	Liu *et al.*^[44]^ (2020)
	ASCs	PureExo® Exosome isolation kit	USPIO	5 µg and 25 µg/100 µL PBS	C57BL/6 mice	IM	After injection	Busato *et al.*^[45]^ (2016)
SPECT	Human umbilical vein endothelial cells	UC	^99m^Tc-AnnV	2.0 ± 0.5 × 106 LEVs/2.0 ± 0.4 MBq/150 µL	BALB/c mice	IV	30 min	Giraud *et al.*^[48]^ (2022)
	Plasma	UC	^99m^Tc-HYNICDuramycin	12 ± 3 MBq/100 μL	C57BL/6	IV	1 h	Németh *et al.*^[49]^ (2021)
	HCT116 cells, ASCs	UC	^99m^Tc-TEx-Cy7,^99m^Tc-AEx-Cy7	37 MBq/200 µL PBS	Tumor-bearing mice	IV	1 h, 6 h, 12 h, 18 h, 24 h	Jing *et al.*^[50]^ (2021)
	Melanoma cells(B16F10)	Sucrose density cushion/UC	^111^Indium	1 × 10^11^ particles/mouse	C57BL/6 mice,NSG mice	IV	1 h, 4 h ,24 h	Faruqu *et al.*^[51]^ (2019)
	Erythrocyte	UC	^99m^Tc-tricarbonyl	15 ± 2 MBq/200 μL volume	BALB/c mice	IV	1 h	Varga *et al.*^[52]^ (2016)
	Raw 264.7 cells, HB1.F3 cells	Iodixanol gradient/UC	^99m^Tc-HMPAO	7.4-14.8 MBq, 11.1 MBq of 29-64 μg cells	BALB/c mice	IV	After injection	Hwang *et al.*^[53]^ (2015)
PET	4T1 cells	UC	^64^Cu--NOTA,^64^Cu--NOTA-PEG	50 μCi/mouse, 200 μL	BALB/c mice	IV	1 h, 4 h, 24 h	Shi *et al.*^[54]^ (2019)
PAI	MIA-PaCa-2 cells	UC	Ce6-R	100 μg/mL exosomal protein and 38.4 μg/ml Ce6	BALB/c mice	IV	6 h	Jang *et al.*^[58]^ (2021)
	Tumor cells	China RIBOBIO Biotechnology Co., Ltd.	Au Nanostars and TDSP	200 μg/mL in 100 μl PBS	BALB/c mice	IV	24 h	Zhu *et al.*^[59]^ (2020)
	NIH3T3 cells,CNE-2 cells	Facile chemical oxidation and exfoliation method	DiR	100 μg/mL in 100 μL volume	BALB/c nude mice	IV	2 h, 4 h, 8 h	Ding *et al.*^[60]^ (2019)
Multimodal imaging	4T1 cells, HeLa cells, Raw 264.7	UC	CDs:Gd,Dy-TAT	30 mg·kg-1·wt	BALB/c mice	IV	6 h, 12 h, 24 h	Yang *et al.*^[64]^ (2021)
	HepG2 cells, L02 cells	UC	Ag ,Fe_3_O_4_	20 μg /mL	-	-	12 h	Tayyaba *et al.*^[65]^ (2020)
	Expi293F cells, HepG2 cells	Iodixanol gradient/UC	DiR,mCherry, ^111^indium-DTPA	1 × 10^11^ cells in 200μL PBS; 5-10 MBq; in 200 μL	BALB/c mice	IV	24 h; 0-30 min, 4 h, 24 h	Lázaro-Ibá́ez *et al.*^[66]^ (2021)
	293T cells, C3H strain HCA1 cells	Sucrose density cushion/UC	PalmGRET	100 µg	C3H/HeNCrNarl mice	IV	5 min, 10 min, 20 min, 30 min	Wu *et al.*^[67]^ (2020)

BLI: Bioluminescence imaging; TFF: Tangential flow filtration; MSC: mesenchymal stromal cell; NMRI: Naval Medical Research Institute; SEC: size exclusion chromatography; IV: intravenous; IP: Intraperitoneal injection; FI: Fluorescence imaging; MRI: Magnetic resonance imaging; UC: ultracentrifugation; CESC: cartilage endplate stem cell; CEP: cartilage endplate; IVDD: intervertebral disc degeneration; MSC: Mesenchymal stem cell; DiD and DiR: near-infrared dyes; SPECT: Single photon emission computed tomography imaging; IM: intramuscular injection; ASCs: adipose stem cells; USPIO: ultrasmall superparamagnetic iron oxide nanoparticles; PET: Positron emission tomography; PAI: Photoacoustic imaging; NOTA: bifunctional chelator 1,4,7-triazacyclononane-1,4,7-triacetic acid; PEG: polyethylene glycol; Ce6-R: Chlorin e6-loaded R; TDSP: Tumor cell-derived stellate plasmonic.

## BIOLUMINESCENCE IMAGING

Bioluminescence imaging (BLI) mainly refers to the integration of the luciferase gene into the chromosomal DNA of the target cells, so that the cells can secrete luciferase-containing exosomes, and the luciferase gene can be stably expressed in the cell without loss. The most commonly used reporter systems for bioluminescence imaging are fluorescent protein and luciferin/luciferase bioluminescence systems^[27]^.

Hikita *et al.* labeled exosomes with Antares2 and observed long-term accumulation of exosomes *in vivo* using bioluminescence resonance energy transfer (BRET). Implantation of prostate cancer cells expressing CD63-Antares2 into mice facilitated the quantification of exosomes originating from the primary tumors entering the bloodstream. Additionally, it enabled the visualization of the extended homing patterns of exosomes to their respective target organs or tissues over an extended period^[28]^. Gupta *et al*. investigated the bioluminescent tagging of EVs by employing various luciferase enzymes attached to CD63. Their findings revealed that the dispersion pattern of EVs is influenced by the injection route. Moreover, distinct subpopulations of EVs exhibited variations in their biodistribution patterns^[29]^. Luo *et al.* established a genetic mouse model incorporating the Nano-luciferase (NanoLuc) reporter, which was fused with the exosome surface marker CD63 to facilitate exosome labeling. The induction of CD63NanoLuc reporter expression was achieved through tamoxifen. Luciferase assays and bioluminescent imaging outcomes demonstrated the targeted labeling and discernible tissue distribution of naturally occurring exosomes released from cardiomyocytes^[30]^. Sung *et al*. introduced a stabilizing mutation, M153R, in the pHluorin moiety and released exosomes *in vivo*. The incorporation of a non-pH-sensitive red fluorescent tag in the exosomes enabled the observation of the complete exosome lifecycle. This included visualizing processes such as MVB trafficking, MVB fusion, exosome uptake, and endosome acidification^[31]^.

Bioluminescence belongs to self-luminescence and does not require external light source excitation, thus avoiding the interference of natural fluorescent substances^[32]^. At the same time, because there is almost no endogenous light source in mammalian cells and tissues, BLI has extremely high sensitivity, specificity, and good signal-to-noise ratio. In addition, the bioluminescence system has good biocompatibility, and has non-phototoxicity caused by the excitation light during the imaging process, and it can be non-invasive, continuous and dynamic monitoring of various biological processes in the living body in real time^[33-35]^. However, BLI also has its own shortcomings: for example, the emitted fluorescence will be scattered and absorbed when propagating in the tissue, photons will be refracted when they meet the cell membrane and cytoplasm, and different types of cells and tissues have different ability to absorb photons. Therefore, the resolution of BLI will decrease.

## FLUORESCENCE IMAGING

Fluorescence imaging (FI) refers to the implantation of experimental materials (such as nanoparticles, drugs, genes, *etc.*) labeled with fluorescent dyes into animals, and then use external excitation light to excite the materials with fluorescence. The *in vivo* imaging system collects the emitted light signals generated by the fluorescent dyes in the body.

There are two main types of fluorescent dyes commonly used for tracking EVs *in vivo*. One type is lipid dyes (such as PKH67, DiD, DiR, MEMGlow, *etc*.), which have different excitation and emission wavelengths and can effectively label EVs^[33-35]^. The other type of fluorescent dye is a membrane-permeable compound, such as carboxyfluorescein succinimidyl ester (CFSE), which is a lipophilic fluorescent dye that can be passively diffused into cells^[36,37]^. It is colorless itself, but after entering the cell, it removes the acetate group to generate carboxyfluorescein succinimidyl ester (green fluorescence), which is also a commonly used method for effectively tracking EVs^[37]^.

González *et al*. documented that the secure attachment of commercially available fluorescent dyes to exosomes derived from both milk and cancer cells (specifically U87 and B16F10 cancer cells) surfaces, facilitated by covalent binding, did not alter the inherent physicochemical characteristics of the exosomes^[38]^. The exosomes, now fluorescently labeled, were effectively visualized using *in vivo* optical imaging^[39]^. Xu *et al*. engineered exosomes derived from mesenchymal stem cells (MSC-EXOs) by incorporating DiR, followed by intravenous injection into mice experiencing ischemic stroke. The near-infrared fluorescence (NIRF) images obtained indicated the migration of MSC-EXOs into the brains of mice with ischemic stroke, showcasing their ability to elicit therapeutic effects against this condition^[40]^. Song *et al*. developed exosomes labeled with DBCO-Cy5 (Cy5-Exo) derived from cancer cells. The donor cancer cells were subjected to treatment with tetraacetylated N-azidoacetyl-D-mannosamine (Ac4ManNAz), and the resulting azide groups were labeled using near-infrared fluorescent dye-conjugated dibenzylcyclooctyne (DBCO-Cy5). Subsequently, non-invasive tracking and imaging of Cy5-Exo were conducted through near-infrared fluorescence (NIRF) imaging in mice with tumors^[41]^.

The advantage of FI is that it has more selectivity of fluorescent probes, flexible labeling methods, and wide wavelength selectivity of fluorescent probes^[29-31]^. However, due to the scattering and absorption of light in biological tissues, coupled with the significant interference of tissue autofluorescence, the resolution and contrast of *in vivo* fluorescence imaging of the visible light spectrum (wavelength 400-700 nm) will decrease as the depth of the tissue increases, and resulting in a low signal-to-background ratio (SBR), and affecting the sensitivity and depth of detection.

## MAGNETIC RESONANCE IMAGING

Magnetic resonance imaging (MRI), commonly known as spin imaging, is an advanced diagnostic modality that utilizes the principles of nuclear magnetic resonance to intricately visualize the internal structures within the body. The principle of MRI is that when there is an external magnetic field, radio frequency waves will act on the protons rotating around the magnetic field to increase its precession angle. When the radio frequency waves stop running, the protons will release the same signals, which are processed by a computer and converted into two-dimensional images to help the staff analyze the state of the target. Using special contrast agents or molecular probes, MRI is used to study cell activities in organisms, including proliferation, differentiation, migration, and aggregation. Superparamagnetic iron oxide (SPIO) nanoparticles exhibit biocompatibility and have demonstrated enhanced targeting capabilities when incorporated into EVs. Notably, ultrasmall SPIO (USPIO), with a size of less than 50 nm, is particularly advantageous for labeling nanoscale EVs owing to their diminutive size^[42]^.

Luo *et al*. conducted an assessment of *in vivo* exosomes derived from normal cartilage endplate stem cells (CESC), differentiating between normal (N-Exos) and degraded exosomes (D-Exos). This evaluation was performed under the influence of intervertebral disc degeneration (IVDD) and apoptosis of nucleus pulposus cells (NPC), utilizing MRI as the diagnostic tool. The results indicated that N-Exos was more conducive to autophagy activation than D-Exos^[43]^. Liu *et al*. engineered a hybrid protein that combined the ferritin heavy chain (FTH1) with a truncated lactadherin. Utilizing FTH1 as an MRI reporter, their investigation revealed that exosomes labeled with FTH1 were observable both *in vitro* and *in vivo* through MR imaging^[44]^. Busato *et al*. introduced an innovative technique for tagging EVs derived from adipose stem cells (ASCs) using USPIO. The MRI detection limit for EVs was determined to be 3 µg *in vitro* and 5 µg *in vivo*, showcasing the effectiveness of this labeling approach^[45]^.

Leveraging MRI for monitoring EVs offers numerous advantages, including its non-destructive, non-invasive, and non-ionizing radiation nature. MRI provides high soft tissue contrast, exceptional spatial resolution (sub-millimeter scale), and overcomes penetration depth limitations, presenting a wealth of information in the resulting images. MRI can be used not only to display the anatomical structure and morphology of tissues and organs, but also to analyze the physiology and biochemistry, tissue metabolism, organ function, *etc*. in the organism in a multi-dimensional and all-round way^[46,47]^. MRI also has some limitations of its own. A low signal-noise ratio (SNR) generally requires signal accumulation to increase SNR, which further reduces the imaging speed. Slow imaging speed can also cause problems such as insufficient time resolution of functional imaging in applications and easy to be affected by motion artifacts.

## SINGLE PHOTON EMISSION COMPUTED TOMOGRAPHY

Single photon emission computed tomography imaging (SPECT) uses single-photon radionuclides as the detection object. The principle of SPECT imaging is to label exosomes with radioisotopes with a short half-life. After the exosomes are injected into the body and reach the expected position, they will emit gamma photons due to their own radioactive decay, which are located on the outer gamma camera probe. The sensitive point of the photon will detect the incoming photons, which will be converted into electrical signals by the photomultiplier tube and amplified at the same time. The amplified signals will form pulse signals. Combined with computer processing, the distribution of exosomes can be displayed in different gray scales or color scales. The probes used for tracing in living cells are mainly labeled with two radionuclides: ^111^In and ^99m^Tc.

Giraud *et al*. used microSPECT/CT imaging to track radiolabeled endothelial EVs and quantify their whole-body distribution *in vivo*^[48]^. Németh *et al*. used SPECT/CT to analyze the biodistribution of Tc-HYNIC-Duramycin labeled EVs in mice, and detected an elevated circulating EV number after the high-fat diet^[49]^. Jing *et al*. employed ^99m^Tc labeling to create radioactive exosomes derived from adipose stem cells, known as Tc-TEx-Cy7, utilizing a hydrophobic insertion mechanism. SPECT imaging results demonstrated that, in comparison to radio-labeled exosomes from adipose stem cells (Tc-AEx-Cy7), Tc-TEx-Cy7 exhibited notably enhanced tumor accumulation in mice with tumors^[50]^. Faruqu *et al*. employed two distinct methods for radiolabeling B16F10-derived exosomes (Exo): intraluminal labeling involving the entrapment of Indium tropolone shuttling, and membrane labeling through chelation of Indium covalently attached to the bifunctional chelator DTPA-anhydride. The results obtained from whole-body SPECT-CT imaging indicated that membrane-labeled Exo exhibited superior radiolabeling efficiency and radiochemical stability compared to intraluminal-labeled exosomes^[51]^. Varga *et al.* used the ^99m^Tc-tricarbonyl complex labeling erythrocyte-derived EVs. The result of SPECT imaging showed that intravenously injected ^99m^Tc-labeled EVs mainly accumulate in the liver and spleen^[52]^. Hwang *et al*. labeled nanovesicles mimicking macrophage-derived exosomes (ENVs) with ^99m^Tc-HMPAO. Upon examining SPECT/CT images of mice injected with ^99m^Tc-HMPAO-ENVs, noticeable higher uptake was observed in the liver, while there was no discernible uptake in the brain^[53]^.

## POSITRON EMISSION TOMOGRAPHY

Positron emission tomography (PET) uses radionuclides as imaging agents to label exosomes, and then uses detectors to accept photons released during the decay of the positron tracer nuclide to form tracer distribution images, thereby reflecting the presence of exosomes in the body Distribution.

Shi *et al*. employed *in vivo* PET to non-invasively monitor copper-^64^Cu-radiolabeled polyethylene glycol (PEG)-modified exosomes. This approach resulted in outstanding imaging quality and facilitated quantitative measurements of both blood residence and tumor retention^[54]^.

SPECT and PET have high specificity in the monitoring of exosomes, and are easy to operate, can quickly obtain semi-quantitative data, and have good accuracy^[55,56]^. Compared with SPECT, PET imaging has higher sensitivity and resolution. However, it also has disadvantages, such as the need to inject radionuclides and the large radiation dose during the inspection.

## PHOTOACOUSTIC IMAGING

Photoacoustic imaging (PAI) is an emerging low-cost, non-invasive imaging technology based on the absorption of light by biological tissues. This method involves capturing images by detecting acoustic signals generated from light absorption, making it an economical and minimally intrusive approach to imaging. Upon exposure to a pulsed laser, biological tissues absorb energy, undergo expansion, and generate pressure changes, thereby initiating the photoacoustic effect. This phenomenon involves the conversion of absorbed light energy into acoustic waves, contributing to a nuanced understanding of biological structures through photoacoustic imaging. The acoustic signal generated by the photoacoustic effect is called the photoacoustic signal. Under the condition of the same light source parameters, the intensity and frequency spectrum of the photoacoustic signal are closely related to the optical, thermal, and elastic properties of the tissue. Photoacoustic imaging technology obtains tissue structure and biochemical information by detecting photoacoustic signals, and realizes functional imaging while reconstructing the image of tissue structure for disease diagnosis and tissue evaluation.

Nguyen *et al*. successfully implemented a pH-responsive PAI-guided chemo-acoustic kinetic combination therapy. This was accomplished by incorporating indocyanine green (ICG), paclitaxel (PTX), and sodium bicarbonate (SBC) into EVs. High-resolution PA imaging showed that SBC-EVs (ICG/PTX) preferentially accumulated in tumor-bearing mice^[57]^. Jang *et al*. engineered re-assembled exosomes derived from tumors (R-Exo) by combining them with a chlorin e6 photosensitizer. The resulting chlorin e6-loaded re-assembled exosomes (Ce6-R-Exo) were observable through photoacoustic imaging and demonstrated the efficient generation of reactive oxygen species within tumor cells when subjected to laser irradiation^[58]^. Zhu *et al*. presented stellate plasmonic exosomes derived from tumor cells (TDSP-Exos). These specialized exosomes were created through the incubation of tumor cells with gold nanostars. Notably, TDSP-Exos demonstrated significant accumulation within deep tumor tissues and displayed impressive performance in the realm of PAI^[59]^. Ding *et al.* engineered nanozyme vesicles with an exosome-like structure through the biomimetic functionalization of GQDzyme/ABTS nanoparticles. The outcomes underscored the potential of GQDzyme/ABTS-based exosome-like nanozyme vesicles as an optimal nanoplatform for advancing *in vivo* deep-tissue tumor-targeted catalytic PAI^[60]^.

Photoacoustic imaging technology combines optical and acoustic imaging with complementary advantages. Photoacoustic imaging has good spatial resolution. Only a very low electromagnetic radiation energy density is required to obtain a photoacoustic signal with a higher signal-to-noise ratio, thereby avoiding the ionization damage caused by high-intensity electromagnetic radiation to biological tissues^[60-63]^. At present, the photoacoustic imaging technology still has certain limitations. First of all, photoacoustic imaging technology uses sound waves as a carrier for imaging. Therefore, it is urgent to improve the imaging effect of bones, air-containing cavities, and other tissues where the transmission of sound waves is blocked. Thirdly, the sensitivity of the ultrasonic transducer needs to be improved to strengthen the detection of deep tissue signals and increase the imaging depth. At the same time, it is also necessary to further improve the imaging resolution.

## MULTIMODAL IMAGING

There are different advantages and disadvantages in tracing *in vivo* cells for different molecular imaging technologies. The single imaging technology to trace EVs *in vivo* has some shortcomings, such as low labeling efficiency, loss of imaging probes, large signal monitoring errors, *etc*. Therefore, the development of new multi-functional probes and the use of multi-modal imaging technology can overcome the shortcomings of a single imaging technology, trace the EVs in the body from multiple aspects, and provide more information.

Yang *et al.* developed a multifaceted engineered nanoplatform using rare earth element Gd and Dy-doped carbon dots (CDs:Gd,Dy) modified with TAT peptide. These were encapsulated into exosomes engineered with RGD peptide (Exo-RGD). The application of this nanoplatform enabled the detection of tumor sites through MRI/CT imaging in mice bearing tumors^[64]^. Tayyaba *et al*. employed HepG2 cancer cells to facilitate the *in situ* biosynthesis of nanoclusters (NCs) composed of silver and iron oxide derived from their respective salts (AgNO_3_ and FeCl_2_). The self-assembled, biosynthesized silver and iron NCs were efficiently loaded onto exosomes as cargo. These silver NCs displayed potential as a fluorescent probe, while the iron oxide (Fe_3_O_4_) NCs served as a contrast agent for both CT and MRI^[65]^. Lázaro-Ibáñez *et al*. conducted an assessment of the impact of five distinct optical and nuclear tracers on the *in vivo* biodistribution of EVs. They presented a comprehensive comparison encompassing fluorescent, bioluminescent, and radioactivity methodologies. Dual labeling of EVs from Expi293F cells can be achieved by noncovalent fluorescent dyes (DiR), covalent modification of ^111^ indium-DTPA, or bioengineering of fluorescent (mCherry) fused with EVs-tagged CD63 or bioluminescent proteins (Firefly and NanoLuc luciferase). The outcomes highlighted that radioactivity emerged as the most precise approach for tracking EVs^[66]^. Wu *et al.* developed PalmGRET, a reporter based on BRET. This innovative creation enables the visualization, tracking, and quantification of EVs across a spectrum of resolutions, from whole-animal to nanoscopic levels, through diverse imaging modalities, including bioluminescence, BRET, and fluorescence^[67]^.

The pivotal aspect for tracing the *in vivo* biological behavior of EVs lies in the development of a quantitative analysis method that boasts high sensitivity and specificity. Combining multiple detection markers into one molecule can provide complementary imaging information. Multimodal imaging-specific molecular markers have attracted the attention of researchers because they can integrate the advantages of multiple imaging methods. Targeted multimodal imaging and theoretical methods are gradually becoming research hotspots, which can provide *in vivo*, non-invasive, quantitative, precise, specific, and longitudinal monitoring methods for the tracing of EVs *in vivo*. The development of multimodal imaging and specific molecular markers will be the future research direction.

## CONCLUSION

The continuous and non-invasive labeling of EVs helps us to understand the biological characteristics of EVs after entering the organism, such as migration, distribution, and survival, as well as the mechanism by which EVs mediate the recovery of cell or tissue function. The development of EV therapy for these diseases has long-term guiding significance. Efficiently monitoring EVs within living organisms poses a challenge that demands reliable techniques ensuring the preservation of membrane integrity and biological activity post-labeling with exogenous probes. Currently, various non-invasive imaging methods are at our disposal for tracking EVs within living organisms, each carrying its unique set of advantages and drawbacks. Table 2 summarizes the advantages and limitations of each method. MRI has a high spatial resolution, but its labeling specificity is still lacking, and the signal intensity may decrease as the cells proliferate. BLI/FI has a lower cost and higher sensitivity than MRI, and can support long-term real-time tracking of cells, but its spatial resolution is not as good as MRI. As the depth of the tissue increases, the intensity of the light signal decreases. SPECT/PET also has a high degree of sensitivity and can continue to detect signals during cell division. However, if researchers are exposed to radioactive internal radiation, the safety needs to be further explored and studied.

**Table 2:** Comparison of various *in vivo* imaging techniques for EVs

**Imaging technology**	**Emission source**	**Detection probe**	**Spatial resolution**	**Time resolution**	**Depth of imaging**	**Sensitivity**	**Costs of experiments**
BLI	near-infrared light source	Fluorescent protein reporter genes	Low	High	Low	High	Low
FI	laser	Fluorescent dyes	Low	High	Low	High	Low
MRI	radio-frequency pulse	Superparamagnetic iron oxide (SPIO); ultrasmall SPIO (USPIO, < 50 nm)	High	Low	High	Low	High
SPECT	γray	radioisotope	Low	High	High	High	High
PET	γray	radioisotope	Low	High	High	High	High
PAI	laser	nanoparticles	Low	High	High	High	Low

BLI: Bioluminescence imaging; FI: Fluorescence imaging; MRI: Magnetic resonance imaging; SPECT: Single photon emission computed tomography imaging; PET: Positron emission tomography; PAI: Photoacoustic imaging.

At present, there is a scarcity of imaging platforms specifically designed for the dynamic tracking of EVs at the *in vivo* level. The present optical imaging, magnetic resonance imaging, and nuclide imaging platforms rely on cellular tracers or medical imaging equipment. Attempts to achieve compatibility with EVs involve adjusting parameters, yet this often results in experimental designs constrained by the imaging threshold of the instrument. Therefore, it is essential to construct a specialized imaging platform for the dynamic tracing of EVs. Due to the lack of imaging platforms dedicated to the dynamic tracing of EVs at the *in vivo* level, we strongly support the utilization of multimodal imaging techniques that offer a wide array of advantages. Multimodal imaging can combine the advantages of different imaging techniques to provide more comprehensive and accurate exosome information. For example, combining optical imaging with magnetic resonance imaging or nuclide imaging can simultaneously obtain information on the location, distribution, and dynamics of EVs. Such an integrated approach will help us to understand the biology of EVs more deeply while providing more effective tools for diagnostic and therapeutic applications of EVs. Therefore, we should actively advocate and carry out research on multimodal exosome tracking imaging.

## DECLARATIONS

### Copyright

© The Author(s) 2023.
